# Low-profile elastic exosuit reduces back muscle fatigue

**DOI:** 10.1038/s41598-020-72531-4

**Published:** 2020-09-29

**Authors:** Erik P. Lamers, Juliana C. Soltys, Keaton L. Scherpereel, Aaron J. Yang, Karl E. Zelik

**Affiliations:** 1 Department of Mechanical Engineering, Vanderbilt University, Olin Hall, 2400 Highland Ave., Nashville, TN 37212 USA; 2 Department of Biomedical Engineering, Vanderbilt University, Nashville, TN USA; 3 Department of Physical Medicine & Rehabilitation, Vanderbilt University, Nashville, TN USA

**Keywords:** Engineering, Biomedical engineering, Mechanical engineering

## Abstract

We investigated the extent to which an un-motorized, low-profile, elastic exosuit reduced the rate of fatigue for six lumbar extensor muscles during leaning. Six healthy subjects participated in an A-B-A (withdrawal design) study protocol, which involved leaning at 45º for up to 90 s without exosuit assistance (A1), then with assistance (B), then again without assistance (A2). The exosuit provided approximately 12–16 Nm of lumbar extension torque. We measured lumbar muscle activity (via surface electromyography) and assessed fatigue rate via median frequency slope. We found that five of the six subjects showed consistent reductions in fatigue rate (ranging from 26% to 87%) for a subset of lumbar muscles (ranging from one to all six lumbar muscles measured). These findings objectively demonstrate the ability of a low-profile elastic exosuit to reduce back muscle fatigue during leaning, which may improve endurance for various occupations.

## Introduction

Prolonged leaning and repeated lifting often lead to fatigue of the low back muscles, specifically the muscles that extend the lumbar spine. Muscles undergo undesired functional changes as they fatigue (e.g., tremoring^1^ and/or sensorimotor deficits^2^), which can result in discomfort, soreness, exhaustion or the inability to produce a desired force^3,4^. Lumbar muscle fatigue can have detrimental effects on task performance and can negatively impact a person’s productivity, satisfaction or safety (e.g., for populations that frequently perform tasks that are demanding on the low back, such as nurses^5,6^, construction workers^7^, automotive assembly workers^8^, and workers in manual material handling environments^9^). Lumbar muscle fatigue can be reduced during leaning or lifting using external aids and wearable assistive devices^10,11^. However, most devices are limited by practical factors such as affordability, form factor, and their ability to integrate into current workflows without interfering^10,12–15^.

To address these practical factors, we previously introduced a new elastic exosuit that combines the low-profile benefits of daily clothing with the physical assistance benefits of an exoskeleton^13,16^. This wearable device can fit underneath typical daily clothing and uses elastic elements to apply assistive forces in parallel with the lumbar extensor muscles to reduce the effort required by these muscles. In more recent prototypes, passive or motorized clutching has been incorporated so that the wearer can engage/disengage assistance on demand^16^. In the current work, we use a force-instrumented prototype, which will hereafter be referred to as the *exosuit*. We previously demonstrated that an early prototype of the exosuit reduced the mean muscle activity of lumbar extensor muscles by 23% to 43% during leaning and 14% to 16% during lifting (*N* = 8^13^). These reductions, along with subjective feedback from prior study participants, suggest that the exosuit may also reduce lumbar muscle fatigue. However, lumbar muscle fatigue was not evaluated in the prior study because it required a different experimental protocol. Moreover, in Lamers et al.^13^, only two lumbar extensor muscles were monitored; however, several muscles contribute to lumbar extension^17^. It is currently unknown to what degree the exosuit can reduce the rate of lumbar muscle fatigue. Furthermore, it is unknown whether these effects are consistent between subjects, or whether these changes in fatigue occur uniformly across multiple back extensor muscles.

The purpose of this study was to evaluate whether the exosuit reduced the fatigue rate of six lumbar extensor muscles during a leaning task, relative to a control condition (i.e., no exosuit assistance). Since we did not know a priori whether individual users would adopt similar muscle activation patterns when receiving exosuit assistance, we approached this study primarily from a subject-specific perspective (i.e., using single-subject study design and analysis methods). Secondarily, we compiled and reported group-averaged results as a supplementary analysis.

## Methods

### Summary

Twelve subjects (seven males, five females) participated in the Day 1 data collection (detailed in “Day 1—measurement validation”). The purpose of Day 1 was measurement validation; to ensure we could objectively measure changes in the rate of lumbar muscle fatigue in individual subjects, using common surface electromyography (sEMG) techniques^18^. Subjects did not wear the exosuit during Day 1, rather subjects performed leaning tasks with heavier vs. lighter hand-held weights to induce higher vs. lower rates of lumbar muscle fatigue, using an A-B-A protocol. First, subjects performed the task while holding a 16 kg mass (i.e., more fatiguing condition—A1), then holding an 11 kg mass (less fatiguing condition—B), then holding the 16 kg mass again (A2). These masses were chosen to induce a contraction magnitude of at least 20% MVC (maximum voluntary contraction), which is considered the minimum contraction level to elicit fatigue that can be detected with sEMG^1,19^. Furthermore, study participants confirmed that they experienced noticeably more back muscle fatigue when holding the 16 kg vs. 11 kg mass, as expected. Six of twelve subjects exhibited a discernible decrease in fatigue rate (based on median frequency slope of sEMG) for a subset of lumbar muscles while holding the lighter mass (11 kg). To come to this conclusion, we visually inspected individual subject results and judged that six of the twelve subjects exhibited reduced median frequency slopes for the 11 kg vs. 16 kg conditions (details in “Day 1—measurement validation”). Thus, for these subjects we had high confidence that we could objectively measure changes in their back muscle fatigue rates. Next, these six subjects (four male, two female, 23.5 ± 1.4 year, 69.6 ± 7.7 kg, 1.8 ± 0.1 m) participated in the exosuit evaluation session (Day 2, detailed in “Day 2—exosuit evaluation”). The purpose of Day 2 was to assess whether the exosuit reduced the fatigue rate for one or more of the six recorded lumbar muscles. Subjects performed leaning without (A1), then with (B), then again without (A2) assistance from the exosuit, in each case while holding the same mass. We then assessed differences in muscle fatigue rate via median frequency slope of sEMG. All subjects reported no history of low back pain in the past six months and gave written, informed consent prior to testing. The experimental protocol was approved by the Vanderbilt University Institutional Review Board, and these methods were carried out in accordance with the IRB-approved protocol.

### Instrumentation and prototype

**Figure 1:**
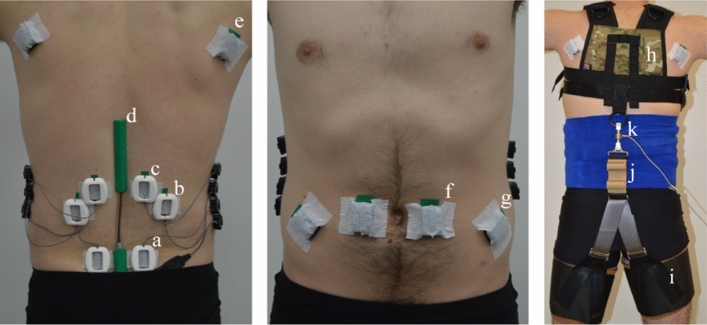
Subject experimental setup (Day 2). sEMG sensors were placed bilaterally over the multifidus muscles (2 cm lateral to the L5 vertebrae, **a**), the iliocostalis lumborum muscles (4 cm lateral to the L2 vertebrae, **b**), the longissimus thoracis muscles (2 cm lateral to the L1 vertebrae, **c**), the latissimus dorsi muscles (scapular level **e**), the rectus abdominis muscles (2 cm lateral to the umbilicus, **f**), and the external obliques (8 cm lateral to the umbilicus, **g**). Each sEMG sensor on the low back was covered with a protective cover to minimize motion artifacts caused by the exosuit moving above the skin and sensors. A digital goniometer (**d**) was adhered to the skin, spanning from the L5 to the L1/T12 vertebrae. A compression wrap (right) was wrapped around the torso to protect sEMG electrodes and prevent wires from snagging. The exosuit prototype was comprised of a trunk harness (**h**) and left/right thigh sleeves (**i**), which were coupled along the backside of the subject via an elastic element (**j**). A uniaxial load cell (**k**) measured forces in-series with the elastic element.

On Day 1, we placed six sEMG sensors (Delsys, Natick, MA, USA) bilaterally over lumbar extensor muscles: right and left lumbar multifidus (RLM and LLM respectively) at L5 level, right and left iliocostalis lumborum (RIL and LIL respectively) at L2 level and the right and left longissimus thoracis (RLT and LLT respectively) at L1 level (Fig. 1a–c)^18^. Two additional sEMG sensors were placed bilaterally over the latissimus dorsi (LD) muscles (Fig. 1e). A goniometer (Biometrics Ltd., UK) was placed across the lumbar region (sacrum to 12th thoracic vertebrae) to measure the sagittal (flexion/extension) angle of the lumbar spine (Fig. 1d). Day 2 involved the same instrumentation as Day 1, but with the addition of four sEMG sensors placed bilaterally over abdominal muscles (rectus abdominis and external oblique, Fig. 1f,g) to provide insight on potential co-contraction of these muscles^20^. All skin surfaces were abraded with alcohol wipes prior to sEMG placement in accordance with SENIAM guidelines^21^. Additionally, plastic guards were placed over each sEMG sensor on the low back (Fig. 1) to minimize motion artifacts due to the movement of the exosuit bands. Abdoli et al.^22^ used similar methods to reduce motion artifact when testing their personal lift assist device.

**Figure 2:**
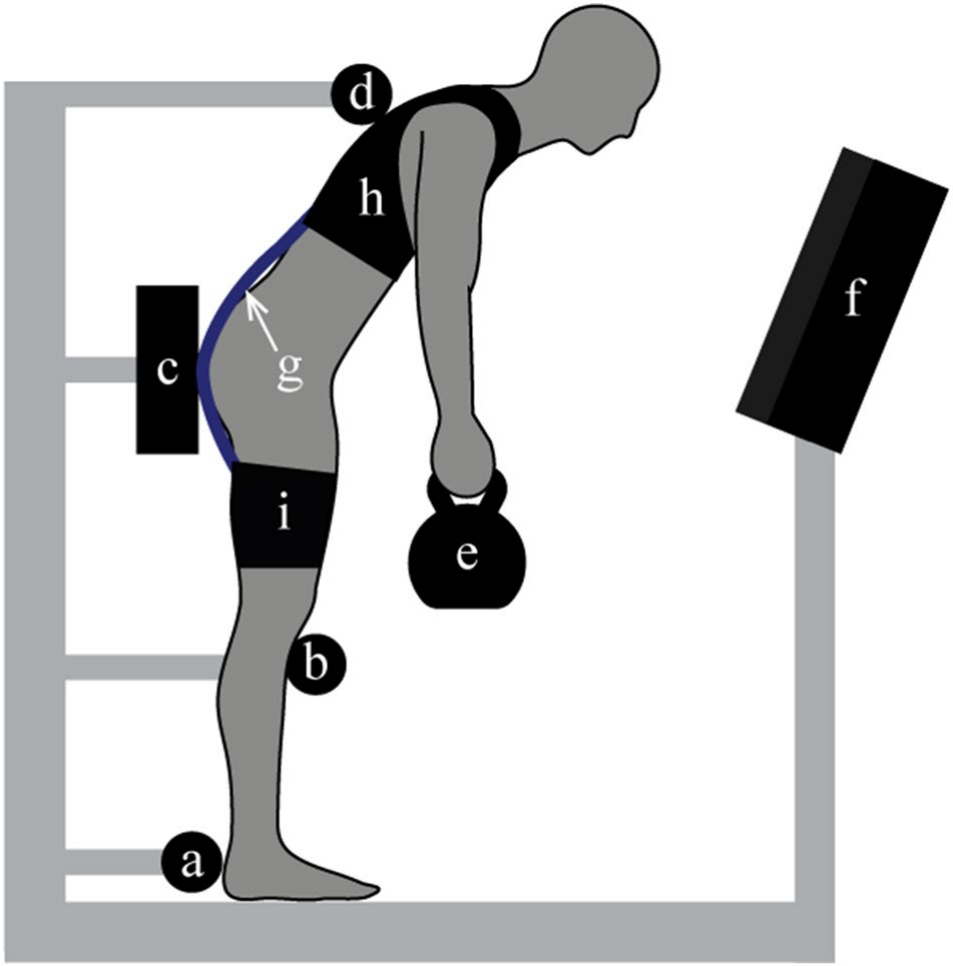
Experimental test setup. During leaning trials, participants stood within the test-apparatus which constrained the position of their feet (**a**), knees (**b**), and pelvis (**c**) with hard stops. A compliant trunk constraint (**d**) allowed subjects to use tactile feedback on their upper back to maintain a consistent trunk angle but minimized their ability to load their lumbar muscles by pushing against the constraint. While leaning forward at 45°, and holding a mass (**e**), subjects were given visual feedback (via lab monitor, **f**) of their lumbar angle and instructed to maintain their preferred lumbar angle (designated prior to trial A1). Subjects were also given visual feedback of their left/right latissimus dorsi muscle activity and were asked to minimize the activity magnitude. During the second trial (trial B) of the Day 2 session, subjects were given lumbar torque assistance via the elastic element (**g**) which ran along their backside, connecting the trunk interface (**h**) to the thigh interfaces (**i**) of the exosuit.

On Day 2, subjects wore an instrumented exosuit prototype that applied an extension torque about the lumbar spine during the intervention trial (trial B in the A-B-A protocol). The prototype, described in detail in previous work^16^, couples a trunk harness to two thigh sleeves with an elastic element (Figs. 1h–j, and 2h,i,g). The magnitude of assistive torque provided by the exosuit was adjusted manually and measured using an in-series load cell (Fig. 1k). The tension for each user was set between 150 and 200 N (the range in tension was due to practical challenges such as exosuit fit). For each subject this resulted in approximately 12–16 Nm of constant torque assistance (assuming a moment arm of 0.08 m between the L5-S1 joint and the exosuit elastic element line of action^13^) throughout the intervention trial. Load cell, sEMG and goniometer data were collected synchronously at 2000 Hz.

### Test apparatus/setup

In order to enable valid comparisons between trials, we developed a protocol to ensure that subjects had the same posture (both full-body and local lumbar angle) for each trial, because lumbar muscle fatigue is sensitive to changes in these postures^23,24^. To ensure subjects adopted the same full-body posture during each trial we developed and used a custom scaffold (Fig. 2) that is similar in function to the postural constraint device in Roy et al.^25^. The scaffold has rigid foot, knee and pelvis constraints that set the position of the lower-body segments (Fig. 2a–c). A compliant trunk constraint (thin, taut string spanning the width of the user) then provides the subject with light tactile feedback on the upper back to help them maintain a consistent trunk angle (Fig. 2d). Note that the string does not provide enough reaction force for the subject to induce non-negligible load on their lumbar muscles when they pushed against it. To ensure subjects adopted the same lumbar spine angle in each trial, we asked subjects to maintain their lumbar angle within ± 2° (which we determined in pilot tests to be a controllable lumbar angle range) of their preferred lumbar angle (established prior to trial A1) using real-time visual feedback. Visual feedback of the lumbar angle (from the goniometer) was displayed on a computer monitor in front of the subject (Fig. 2f).

To prevent subjects from compensating with their LD muscles while leaning, we used real-time visual feedback to minimize LD muscle activity. While the primary function of the LD muscle is to adduct, extend and medially rotate the shoulder joint^26^, it also generates an extension moment about the lumbar spine^27^. In pilot tests, we observed that some subjects would greatly increase their LD activity partway through a leaning trial. This increased activity occurred inconsistently and affected the activity of the six lumbar muscles we sought to study. As a result, this LD behavior confounds our ability to objectively assess lumbar muscle fatigue across trials (e.g., if a subject employed this sporadic strategy for one trial, but not the other trial) and to apply established muscle fatigue analysis methods based on linear regression. To avoid this confound, subjects had real-time visual feedback of both the left and right LD sEMG signals (simultaneously with the lumbar angle feedback), and were asked to minimize LD activity during each trial. See Discussion for more on this topic.

### Study design

During both Day 1 and Day 2 testing sessions we used an A-B-A protocol (withdrawal design) in which we established a baseline fatigue rate (first control—A1), introduced an intervention (B) that we expected to reduce the fatigue rate, and then removed the intervention (second control—A2) to examine if the fatigue rate returned back to the initial baseline rate. We compared the rates of individual muscle fatigue (within and between subjects) for three cases:

- Case I: First control trial (A1) vs. intervention trial (B)
- Case II: Second control trial (A2) vs. intervention trial (B)
- Case III: First control trial (A1) vs. second control trial (A2).

Cases I and II informed whether the intervention (Day 1: lighter held mass, Day 2: exosuit assistance) reduced muscle fatigue relative to the control trials. Case III assessed whether fatigue rates returned to the baseline level, which would strengthen the inference that the observed change was caused by the intervention, rather than chance, task acclimation or natural variability^28^.

### Subject testing

#### Day 1—measurement validation

Subjects first performed maximum voluntary contractions (MVC) of their lumbar muscles in a back-extension machine, by extending their trunk (aligned parallel with the ground) against manual resistance applied by the test administrator. Subjects then performed MVCs for their LD muscles by pulling down on an anchored rope with their left and then right arm (as if attempting a pull-up with one arm). MVC trials were 5 s in duration and were separated by a rest period of at least 1 min. Next, the foot, knee, pelvis and trunk constraints of the scaffold (Fig. 2) were adjusted for each subject. The trunk constraint was adjusted until the trunk angle was approximately 45°. Subjects then assumed a leaning posture and were instructed to choose a preferred lumbar angle while keeping their back flat. This was to ensure that the preferred lumbar angle was near neutral spine alignment, thus mitigating passive torque contributions from spinal ligaments (observed during large magnitudes of lumbar flexion^29^). A shaded band with ± 2° of the preferred lumbar angle was placed on the real-time visual feedback monitor. During testing, subjects were instructed to keep their lumbar angle within this shaded band. Subjects performed the first leaning trial (A1) with a 16 kg mass, the second trial (B) with an 11 kg mass, and the third trial (A2) with a 16 kg mass. Leaning trials were 90 s long, which we determined during pilot testing to be sufficiently long for participants to report considerable back muscle fatigue. Subjects were given at least 15 min in between trials to recover^30^.

The purpose of the Day 1 session was to perform measurement validation on a subject-specific basis. In other words, Day 1 allowed us to confirm (or refute) that for a given subject we could confidently measure differences in back muscle fatigue rate (via sEMG) when comparing tasks with different physical demands (i.e., holding 16 kg vs. 11 kg). This subject-specific measurement validation was considered a critical inclusion criteria because we needed to confirm that subject sEMG median frequency followed logical trends (i.e., indicating more fatigue with higher weights), in order to ensure the fidelity and interpretability of data from the Day 2 session. Subjects only participated in the Day 2 data collection if during Day 1 they exhibited a reduction in lumbar muscle fatigue rate (as estimated by the median frequency slope of sEMG, detailed below in “sEMG fatigue analyses” section) for at least 1 lumbar muscle during the reduced load condition (11 kg, trial B) relative to the higher load conditions (16 kg, trials A1 and A2), and if the remaining lumbar muscles showed small changes (i.e., where the variation between A1 and B or A2 and B was smaller than or similar to variation between A1 and A2). Differences in lumbar muscle fatigue rate were assessed using visual inspection of the sEMG median frequency data and slope (see “sEMG fatigue analyses” section).

#### Day 2—exosuit evaluation

Testing performed during the Day 2 session was similar to Day 1, except that for trial B the subjects (Table 1) were given lumbar torque assistance via the exosuit, rather than a different hand-held weight. Subjects performed the same MVC and setup procedures as detailed in “Day 1—measurement validation” section, with the addition of MVC trials for the abdominal muscles (in which subjects flexed their trunk against manual resistance while positioned face up in the back-extension machine). Subjects then performed the first leaning trial (A1) without exosuit assistance, the second trial with assistance (B), and the third trial again without assistance (A2). Subjects held the same mass for each trial and rested at least 15 min between trials. Subjects had the exosuit donned for the entire Day 2 session, but the elastic element was fully detached during the control trials (A1 and A2) such that the exosuit provided no assistance. Subject 2 (Table 1) held 20.5 kg, while the remaining subjects held 16 kg (Fig. 2e). Subject 2 exhibited greater resistance to fatigue during Day 1 testing and was given a heavier mass to ensure measurable levels of fatigue within the prescribed trial duration (90 s).

**Table 1:** Individual subject metrics for Day 2 intervention session.

Subject #	Mass (kg)	Height (m)	Age	Gender
1	59.1	1.73	23	F
2	63.6	1.73	24	F
3	68.2	1.68	22	M
4	70.5	1.91	23	M
5	77.3	1.85	26	M
6	79.1	1.8	23	M
Mean ± SD	69.6 ± 7.7	1.8 ± 0.1	23.5 ± 1.4	–

### sEMG fatigue analyses

**Figure 3:**
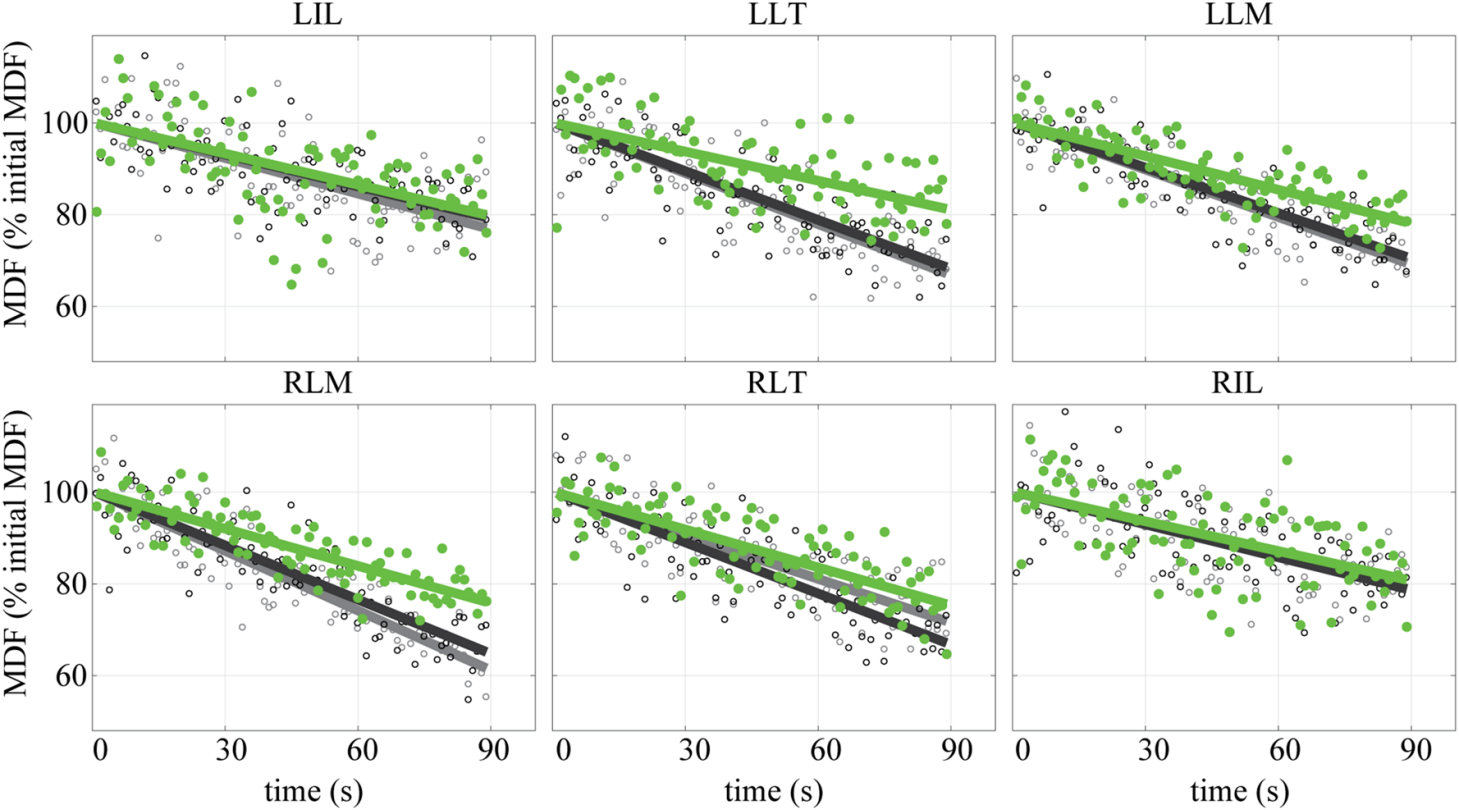
Representative (*N* = 1) muscle-level, median frequencies (Day 2). The decrease in median frequency (MDF) of the sEMG over the duration of leaning is fit by linear regression lines. The slope of the regression line (i.e., MDF slope) is commonly used as a measurement of the rate of muscle fatigue^31^. Data are plotted for each muscle and each trial condition; control trial A1 (black line, open black circles), intervention trial B (green line, shaded green circles), control trial A2 (gray line, open gray circles). This figure illustrates the MDF slope being significantly reduced for some muscles (e.g., LLT, LLM, and RLM) while wearing the exosuit, but not significantly reduced for other muscles (e.g., LIL and RIL). Note that the variability in MDF data points (i.e., each sample point shown) is common and characteristic of this sEMG measurement of fatigue^35,36^. Depicted is data from Subject 3.

Raw sEMG signals were filtered with a fourth-order, zero-lag, bandpass filter (10–500 Hz)^18^. Median frequency (MDF) and the root mean square (RMS) of the filtered sEMG signals were calculated over non-overlapping 1-s epochs for each trial^18,31^ (Fig. 3). Linear regressions were fit to MDF data from each muscle, where the y-intercept of the regression indicated the initial MDF (value at t = 0). Fatigue measurement epochs were discarded from the regression analysis if a subject’s lumbar angle showed sustained deviation greater than ± 2°. We present the slope of the regression fit in terms of the initial MDF value, i.e., in units of % initial MDF per second (hereafter simply referred to as the MDF slope). RMS for each muscle was normalized to the maximum RMS magnitude observed in the MVC trials. In single-subject studies such as this visual inspection is recommended as the primary assessment method whereas inferential statistics are only intended to supplement visual inspection procedures^32^. Therefore, visual inspection was used to assess the effect of the exosuit on single-subject, muscle-level MDF slope. Specifically, two of the research investigators independently assessed whether the intervention (trial B) appeared to have an effect on MDF slope relative to the controls (trials A1 and A2). As a key summary metric, they summed the number of individual muscles across all six subjects that showed reduced MDF slope during trial B. To supplement findings from visual inspection, the effect of the exosuit on single-subject, muscle-level MDF slope was also analyzed via analysis of covariance with unequal slopes (aoctool function in MATLAB). Significant main effects (α = 0.05) from the analysis of covariance, were followed up with a multiple comparison test using a Tukey–Kramer test (multcompare function in MATLAB).

For inter-subject analysis, if the data passed a Shapiro–Wilk normality test (swtest function in MATLAB), then repeated measures analysis of variance tests (rmfit function in MATLAB) were performed to assess changes in MDF slope for Cases I–III. Friedman tests (Friedman function in MATLAB) were performed if data did not pass the Shapiro–Wilk test. Significant main effects (α = 0.05) from the repeated measures analysis of variance and Friedman tests were followed up with a multiple comparison test using a Tukey–Kramer test (multcompare function in MATLAB).

## Results

### Day 1—measurement validation

Six of the twelve subjects who participated in the Day 1 session showed reductions in MDF slope for a subset of lumbar muscles when holding the lighter weight, and only small changes in MDF slope for the remaining lumbar muscles (based on a visual inspection). These six individuals were invited to participate in Day 2. The six remaining subjects exhibited inconsistent muscle fatigue behaviors (e.g., no clear changes in MDF slope, or a combination of increases and decreases in the MDF slope for different muscles) despite differences in held mass. Consequently, we did not have confidence that we could reliably monitor changes in fatigue for this subset of participants. We therefore excluded them from Day 2 exosuit evaluation. Also, of note, sEMG RMS magnitude results on Day 1 were not consistent (i.e., often did not exhibit clear or expected fatigue trends) and sometimes appeared to contradict MDF findings (which has also been observed in previous literature^33^). Since the RMS metric could not be readily interpreted on a subject-by-subject basis, it was not used in the Day 2 study to evaluate fatigue. This analysis approach is consistent with previous literature^25^, in which MDF is typically used as the primary fatigue metric, and RMS is used as a secondary metric or not used at all^34^.

### Day 2—exosuit evaluation (subject-specific)

#### Visual inspection

Two investigators independently reviewed the sEMG results for 36 muscles (6 subjects × 6 lumbar extensor muscles) and determined via visual inspection (Fig. 3) that between 18 and 21 individual muscles showed reduced MDF slope with the exosuit intervention (trial B) vs without the exosuit intervention (trials A1 and A2). See Appendix for subject-specific plots of sEMG MDF (Figs. 8, 9, 10, 11, 12, 13). Abdominal muscle activity was small (< 5% of the MVC magnitudes), and thus was not amenable to fatigue analysis or interpretation using MDF, which requires muscle to reach a higher level of muscle contraction magnitude^1^.

#### Supplementary statistics

##### Case I: First control trial (A1) vs. exosuit intervention (B)

**Figure 4:**
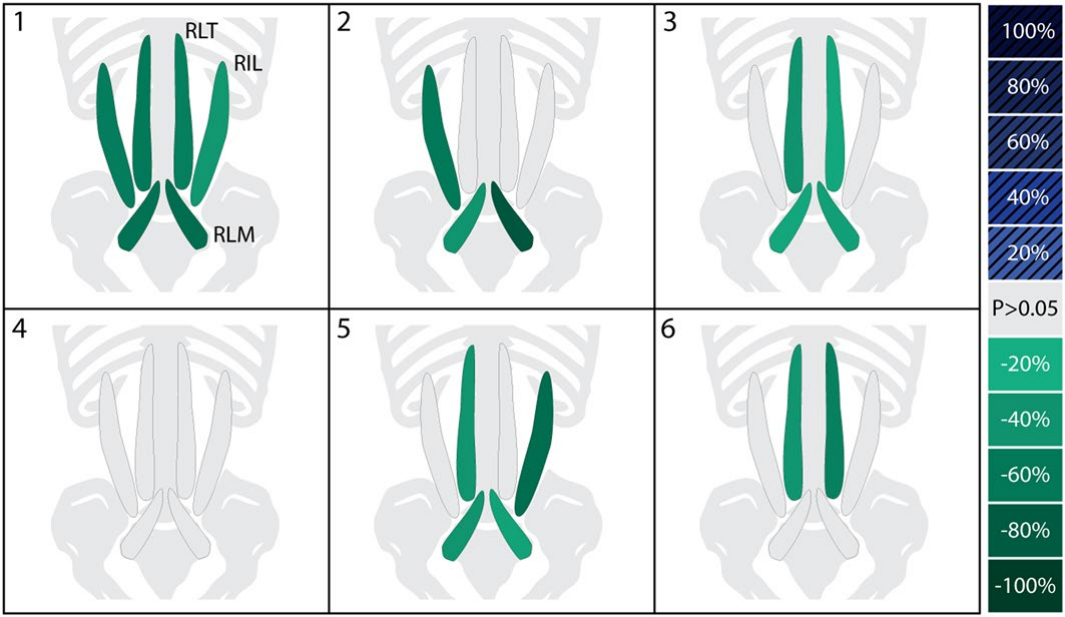
Subject-specific, muscle-level response to exosuit. Depicted are the changes in fatigue rate (MDF slope) for individual muscles when receiving exosuit assistance as compared to not receiving assistance (Case I: A1 vs. B). Significant decreases (and increases) in MDF slope with the exosuit are denoted by green (and blue-hatched) shading, where darker shades indicate larger magnitudes of change. Non-significant changes are denoted by light gray shading. The degree to which individual muscles experienced significant reductions in fatigue varied from subject to subject, with one showing significant reductions for all muscles (e.g., subject 1) and others showing significant reductions for a subset (e.g., subject 2). Shading is based on the analysis of covariance results, which were very similar to visual inspection. These responses highlight the variability across individual subjects, as well as the variability across the individual muscles of a single subject, demonstrating that there are multiple ways a wearer can adapt to and benefit from the exosuit.

Five of six subjects exhibited statistically significant reductions in MDF slope in a subset of muscles (without any significant changes in any other muscles) with the exosuit vs. the first control trial (Table 2, Fig. 4). Of these five, one (subject 1) showed statistically significant reductions for all muscles, and the remaining (subjects 2, 3, 5, 6) showed significant reductions for two to four muscles, and non-significant changes for the remaining muscles. Only one subject (subject 4) showed no significant changes across all muscles. During trial B, subject 5 deviated from the target lumbar posture range (± 2°) at 77 s into the trial, thus the final 13 s of this trial were excluded from analysis.

**Table 2:**
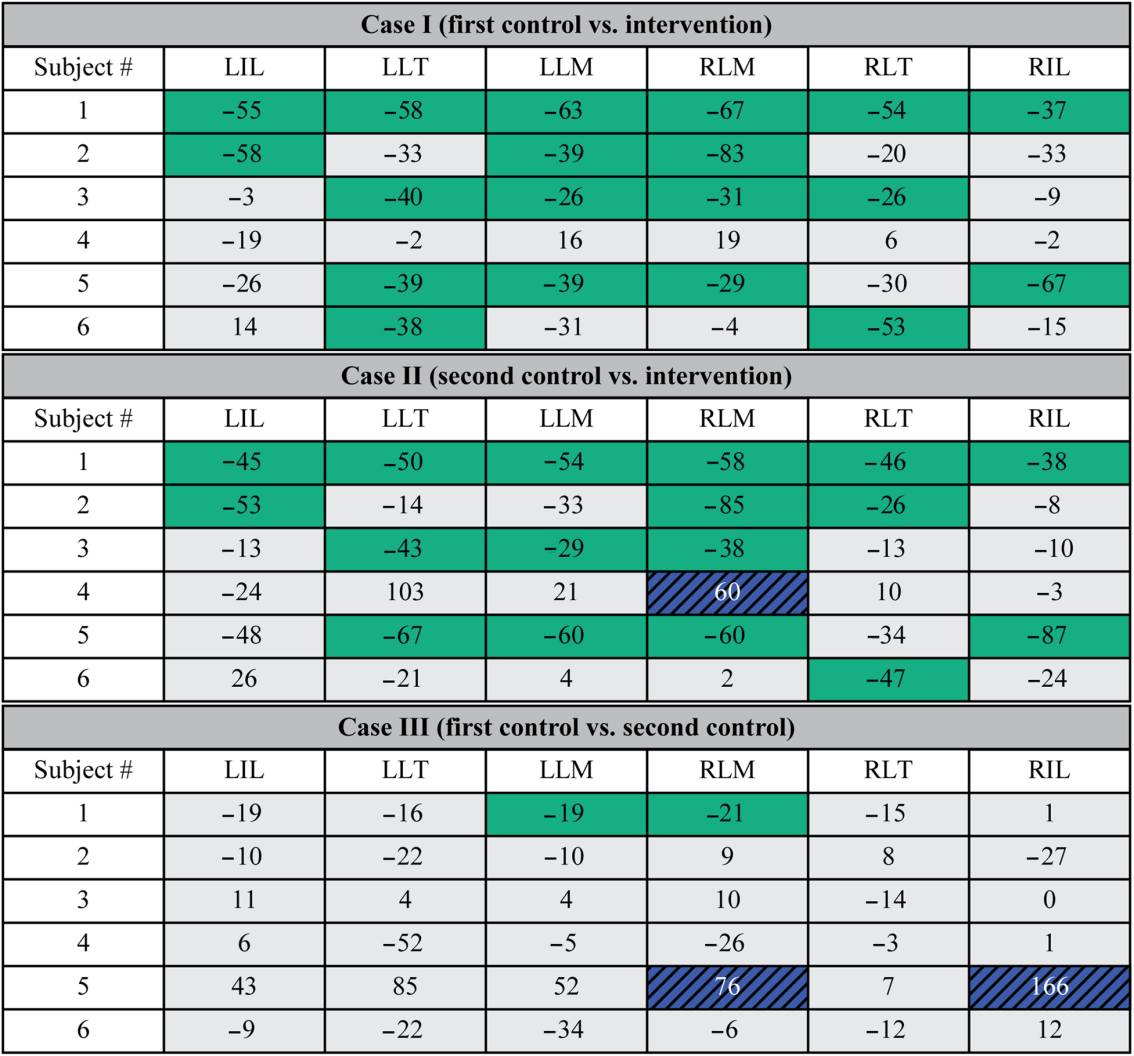
Change in MDF slope for individual muscles across all subjects for Cases I, II and III.

Significant reductions in MDF slope (based on the analysis of covariance) when receiving exosuit assistance are denoted by green shading. Significant increases in MDF slope are denoted by blue shading, and non-significant changes are denoted by light gray shading. Changes are expressed as a percent change in MDF slope from one trial to the next (e.g., Case I, first control vs. intervention) where negative values indicate a reduction in MDF slope when wearing the exosuit relative to the control trials (Case I and Case II). Negative values in Case III indicate a reduction in MDF slope during the second control trial relative to the first control trial.

##### Case II: Second control trial (A2) vs. exosuit intervention (B)

The same five subjects exhibited reductions in MDF slope with the exosuit vs. the second control trial (Table 2), corroborating the results from Case I. One of these five (subject 1) showed significant reductions for all muscles. Four subjects (subjects 2, 3, 5, 6) showed significant reductions for one to four muscles, and non-significant changes for the remaining muscles. Subject 4 showed a significant increase in MDF slope for one muscle, and non-significant changes in the remaining muscles. During trial A2 subject 5 deviated from the target lumbar posture range (± 2°) at 30 s into the trial, thus the final 60 s of data were excluded from analysis.

##### Case III: First control trial (A1) vs. second control trial (A2)

Four of six subjects showed no significant changes in MDF slope for any muscles when comparing the first vs. second control trial (Table 2). Subject 1 showed significant reductions for two muscles, whereas subject 5 showed significant increases for two muscles.

### Day 2—exosuit evaluation (inter-subject)

**Figure 5:**
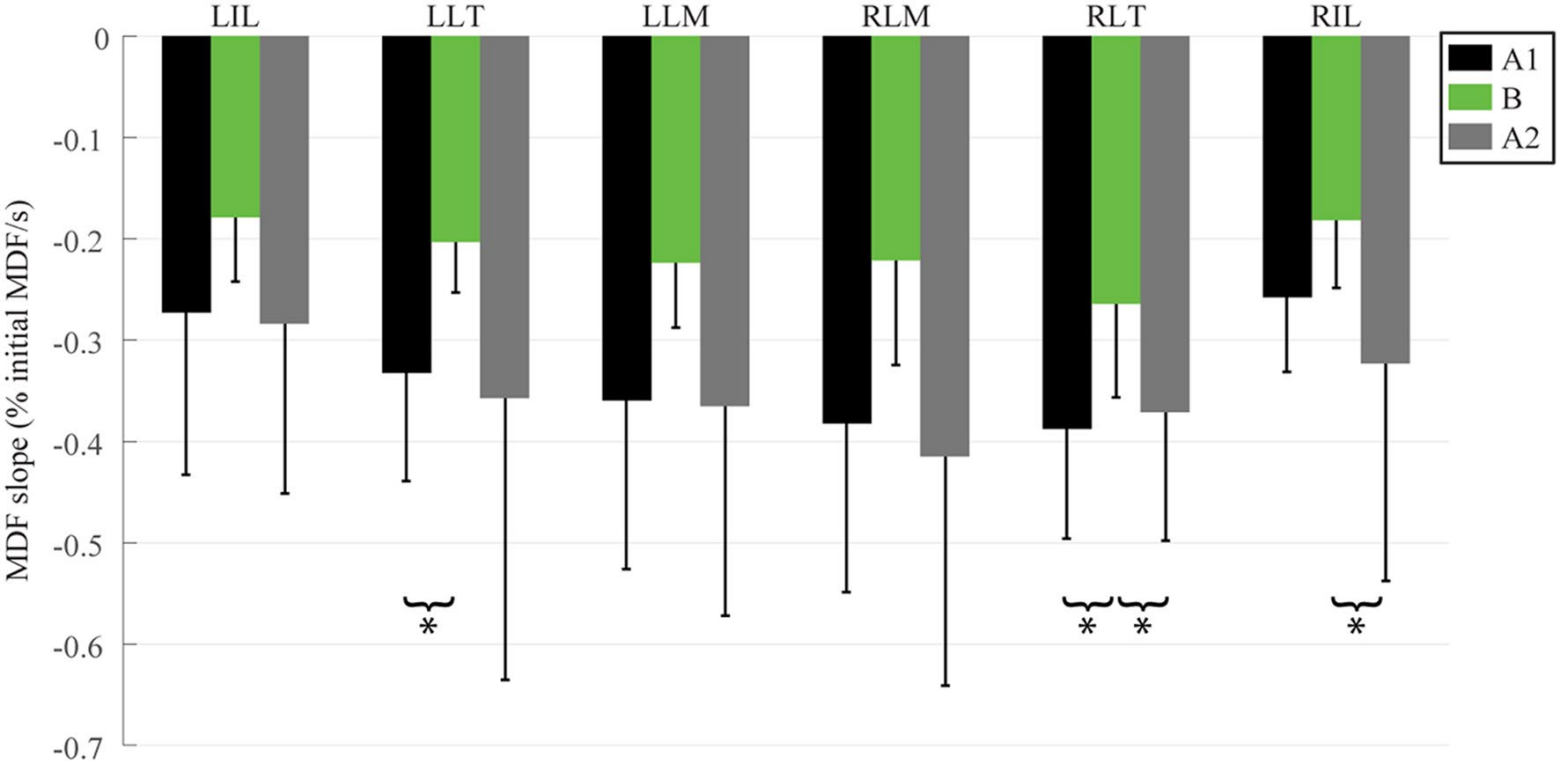
Group-level response to exosuit. The change in percentage slope of the regression line (i.e., MDF slope) is commonly used as an indicator of the percentage change in the rate of muscle fatigue^31^. Reductions in the inter-subject average (*N* = *6*) MDF slopes were statistically significant (indicated by asterisks) for LLT and RLT for Case I (A1 vs. B) and RLT and RIL for Case II (B vs. A2).

On average, across the six subjects during Day 2 (Fig. 5), all lumbar muscles showed mean reductions in MDF slope with the exosuit relative to the first control trial (Case I: LIL: − 34% LLT: − 39% LLM: − 38% RLM: − 42% RLT: − 32% RIL: − 30%) and second control trial (Case II: LIL: − 37% LLT: − 43% LLM: − 39% RLM: − 47% RLT: − 29% RIL: − 44%). Of these muscles, two (LLT and RLT) showed statistically significant reductions in MDF slope (LLT: − 39%, p = 0.020, RLT: − 32%, p = 0.043) with the exosuit versus the first control trial (Case I), and two muscles (RLT and RIL) showed statistically significant reductions in MDF slope (RLT: − 29%, p = 0.047, RIL: − 44%, p = 0.004) with the exosuit versus the second control trial (Case II). Across all subjects in Case I, 19 of 36 muscles showed significant reductions in MDF slope. Similarly, for Case II, 17 of 36 muscles showed significant reductions in MDF slope while only 1 muscle showed significant increases. Between Cases I and II, significant increases in MDF slope were only observed in subject 4. In Case III, only 4 of 36 muscles showed significant changes in MDF slope. No abdominal muscles showed significant changes in MDF slope, nor did abdominal activation exceed 5% of the MVC magnitudes, and thus for brevity these results are not depicted.

## Discussion

### Summary

We found that the exosuit reduced the lumbar muscle fatigue rate during leaning, with benefits exhibited by five of six subjects. The number of muscles that showed fatigue reductions and the magnitude of these reductions varied between individuals. From visual inspection, it was determined that between 18 and 21 individual muscles (of the 36 assessed across all subjects) experienced reduced fatigue rate with the exosuit vs. without the exosuit; and this was corroborated by statistical analysis. Based on inferential statistics, one subject showed significant reductions across all muscles, four showed significant reductions in 1–4 muscles, and one subject showed increases for 1 muscle. Across all subjects, the exosuit significantly reduced the fatigue rates for about half (18 of 36) of the muscles, consistent with findings from visual inspection. These findings add to a growing body of literature (e.g.^13,22^) that indicate that the exosuit provides a viable option for reducing lumbar muscle fatigue in various occupations and work environments where leaning or bending is common (e.g., a doctor performing surgery, a material handler, or a worker at an assembly line).

### To what degree did the exosuit reduce the rate of lumbar muscle fatigue?

Individual subjects showed significant reductions in MDF slope (i.e., fatigue rate) ranging from 26% to 87% across the lumbar muscles (relative to control trials, Cases I and II in Table 2), and exhibited consistent benefits for at least 1 and up to 6 lumbar muscles. Previous studies on lumbar muscle fatigue found that reduced MDF slopes corresponded with increased endurance times (i.e., duration that someone can sustain a leaning posture before task failure)^35^. Thus, this exosuit may have practical applications in work environments where leaning is common, such as dentistry^37^, surgery^38^, nursing^6^, construction^7^ or material handling, and where excessive lumbar muscle fatigue may necessitate workers to take frequent breaks, or may increase the likelihood of mistakes, injuries or employee turnover. For a practical comparison, we processed sEMG data from Day 1 (using the same analysis detailed in “sEMG fatigue analyses” section) and computed fatigue rates for the same six subjects. We found that significant reductions in fatigue rate during Day 1, when holding an 11 kg vs. 16 kg mass, ranged from 21% to 57% across individual muscles; and on average each subject exhibited significant reductions in fatigue rate in 1.4 ± 1.2 muscles. Relative to the Day 2 range (26% to 87% reductions in fatigue rate) and average number of muscles exhibiting reduced fatigue (3.0 ± 1.9 muscles), this suggests that the exosuit assistance provided an offloading effect that was greater than reducing the mass of a weight held about 0.3 m in front of the L5/S1 joint by 5 kg. Stated simply, the exosuit made holding a 16 kg mass less fatiguing on the back than holding an 11 kg mass without the exosuit. These results were nearly identical and the interpretation unchanged even if subject 2 was excluded from the analysis due to lifting a heavier weight on Day 2. This magnitude of benefit is consistent with physics-based estimates, i.e., that the 12–16 Nm assistance from the exosuit (150–200 N applied at 0.08 m moment arm) would be expected to offload 4–5 kg from a hand-held mass located 0.3 m anterior to the L5/S1 joint. Prior studies^22^ and physics based estimates^13^ both indicate that further extending the moment-arm of the device could increase assistance to the user, which may facilitate greater reductions in muscle fatigue; though potentially at the cost of increasing device form-factor (i.e., how much it protrudes from the body).

### Were changes in the lumbar muscle fatigue rate consistent between subjects?

Changes in the rate of lumbar muscle fatigue were not consistent between subjects. Subject 1 showed significant reductions across all lumbar muscles, whereas subjects 2, 3, 5 and 6 had significant reductions for a subset of muscles (ranging from 1 to 4 muscles, Cases I and II). This inter-subject variability may be partially explained by the difference in bodyweight normalized exosuit assistance between subjects (ranging from 2.0 to 3.0 N/kg, Table 3). Subjects who received a higher magnitude of assistance (e.g., subjects 1 and 5 who received 2.7–3.0 N/kg) showed significant reductions across more lumbar muscles than those who received a lower magnitude (e.g., subjects 2, 3 and 6 who received 2.3–2.4 N/kg). These trends suggest that increasing exosuit assistance may yield more significant reductions in lumbar muscle fatigue, as one would intuit. This result is consistent with prior back exosuit studies; for example, Frost et al.^39^ demonstrated that greater magnitudes of torque assistance (generated by using stiffer springs) yielded greater reductions in back muscle sEMG. Subject 4 received the least assistance (2.0 N/kg, Table 3), and did not exhibit reductions in muscle fatigue rate. Aside from receiving less assistance, we are unable to identify (from the data we collected) why this individual responded so differently from the other subjects. We did not observe substantial changes in the antagonistic abdominal muscle activity, which remained small across conditions (i.e., did not exceed 5% MVC), nor did we observe changes in posture; although minor compensatory strategies may have been used which were not captured by our measurements. It is known that people habituate to assistive devices at different rates, and thus differences in learning/adaptation rate may explain some of the inter-subject variability observed in this study; particularly, given the relatively short training/acclimation period. In the future it will be interesting to test subjects after longer (e.g., multi-day or multi-week) training periods, or to test individuals at different time points during the habituation process.

**Table 3:** Subject-specific exosuit assistance magnitude.

Subject #	Assistance (N/kg)	N_I_	N_II_
1	3.0	6	6
5	2.7	4	4
6	2.4	2	1
3	2.3	4	3
2	2.3	3	3
4	2.0	0	0

The magnitude of normalized exosuit assistance (tension in elastic element (N) per body mass (kg)) varied across subjects (ranging 2.0–3.0 N/kg). The number of muscles that showed significant reductions in MDF slope during Case I (N_I_) and Case II (N_II_) tended to be higher for subjects who received greater magnitudes of normalized exosuit assistance.

### Did changes in fatigue rate occur uniformly across the lumbar muscles?

**Figure 6:**
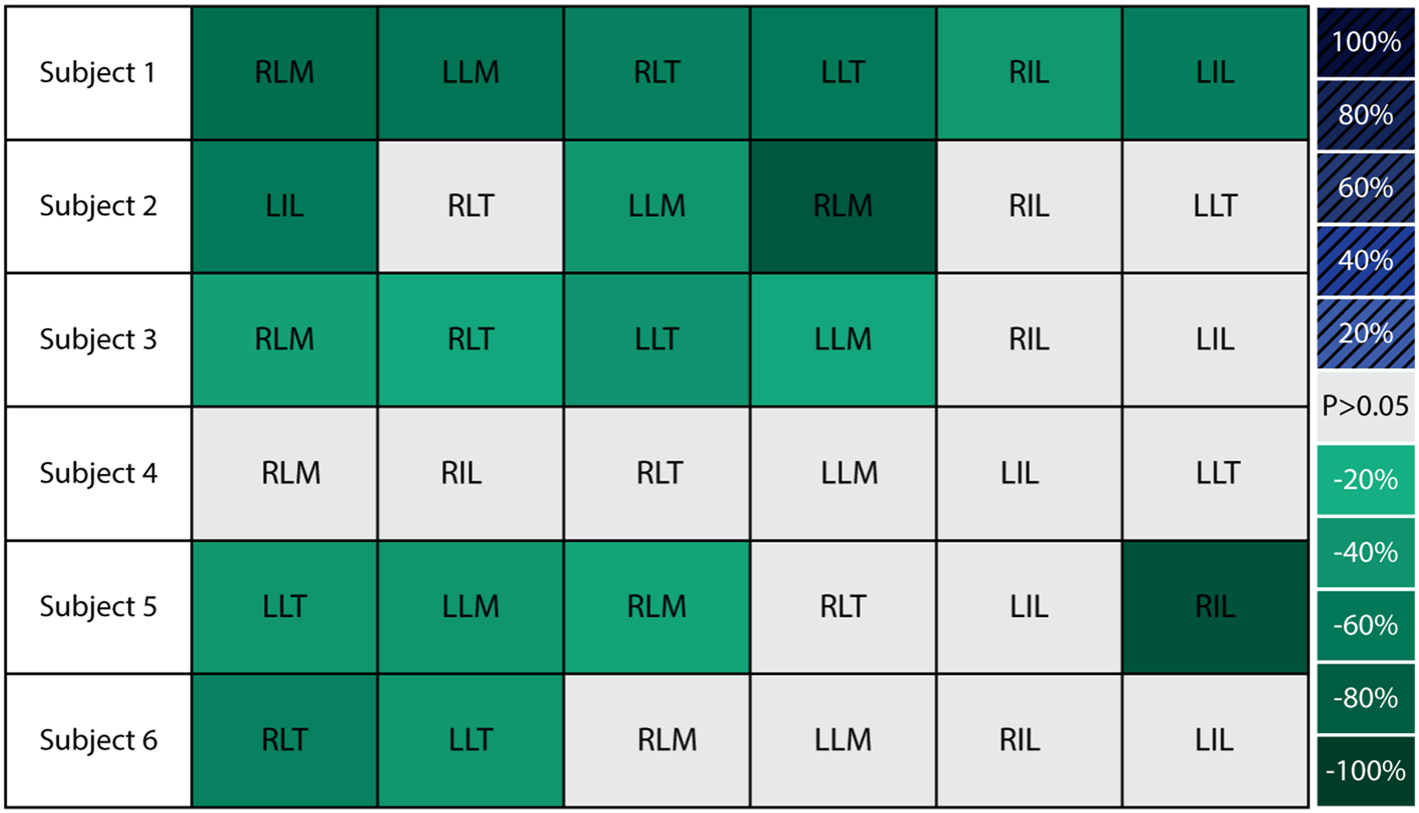
Individual muscle responses to exosuit ordered based on muscle fatigue rate: This shows Case I results (from Table 2) reorganized such that muscles are ordered from left to right, where left is the fastest (and right is the slowest) rate of fatigue during control trial A1. Significant decreases (and increases) in MDF slope are denoted by green (and blue) shading, where darker shades indicate larger magnitudes of change. Non-significant changes are denoted by light gray shading. Muscles that fatigued the fastest during control trial A1 tended to show significant reductions with the exosuit intervention, depicted by the higher density of green boxes on the left (and gray boxes on the right).

Changes in fatigue rate were generally not uniform across lumbar muscles based on findings from both visual inspection and inferential statistics (Table 2, Fig. 4). For instance, subjects 2, 3 and 6 showed considerable variability across muscles; with significant reductions in some muscles and non-significant changes in others (Case I). Even for subjects 1 and 5 who showed significant reductions across 4–6 muscles (Table 2), the magnitudes of these reductions varied across muscles (from 38% to 67% in subject 1, and from 29% to 87% in subject 5). Because many muscles span the lumbar spine, there are countless different muscle activation patterns that can result in the same net lumbar joint torque. This begs the question: how do subjects decide which lumbar muscles to offload/relieve when exosuit assistance is provided? Based on our findings, the lumbar muscles that fatigued the fastest (i.e., had the greatest MDF slope magnitude) during the A1 control trial appeared more likely to have statistically significant reductions when the exosuit intervention was introduced (Fig. 6). This trend suggests that subjects may have chosen to preferentially offload muscles that were fatiguing the fastest during this leaning task. Previous work shows that the fastest fatiguing lumbar muscles best predict endurance time^36^, indicating that they may be a limiting factor for endurance. By preferentially offloading the fastest fatiguing lumbar muscles (as opposed to other, slower fatiguing lumbar muscles), subjects may derive the greatest benefit in terms of increasing their endurance time. The hypothesis that individuals choose to relieve muscles that are fatiguing the fastest, warrants further investigation.

### Insights from group-level analysis

On average, all muscles exhibited reductions in fatigue rate when wearing the exosuit vs. both control trials, by between 29% and 47% (Fig. 5, Cases I and II). However, these reductions only reached statistical significance for the left and right longissimus thoracis muscles for Case I and the right longissimus thoracis and the right iliocostalis lumborum muscle for Case II (Fig. 5). This result (i.e., a limited number of statistically significant reductions) was not surprising given the small sample size (*N* = 6), meaning that the study was underpowered from a group analysis perspective. However, biomechanical interventions often result in high inter-subject variability in responses^28^, which is why we focused on subject-specific findings in this study. Based on the subject-specific results it was clear that individual users received benefits to different lumbar muscles. For instance, subject 2 and subject 6 each benefited from the exosuit yet showed reductions in fatigue for a different (non-overlapping) subset of lumbar muscles (Fig. 4). This observation is potentially relevant for the development of biomechanical test methods or ergonomic assessment standards for exosuits and exoskeletons. For example, if we had only recorded a subset of the lumbar muscles (e.g., only RLM and LLM) we would have concluded that the exosuit benefitted subject 3, but not subject 6 (Fig. 4). But with a more comprehensive sample of lumbar muscles (e.g., the six monitored in this work) we conclude that the exosuit benefitted both subjects 3 and 6; however, these subjects happen to derive benefits for a different subset of back extensor muscles. Thus, assuming that synergistic muscles (e.g., lumbar extensor muscles) will behave or adapt similarly to an intervention may generate misleading conclusions. Given the abundance of muscles that can contribute to lumbar extension it generally will not be known a priori which specific muscles will exhibit benefits for each individual, and thus it may not be known which muscles should be monitored. This may bring about challenges related to the standardization of assessment metrics across tasks and devices, and complicate objective comparisons and interpretations between individuals.

### Latissimus dorsi (LD) observations

**Figure 7:**
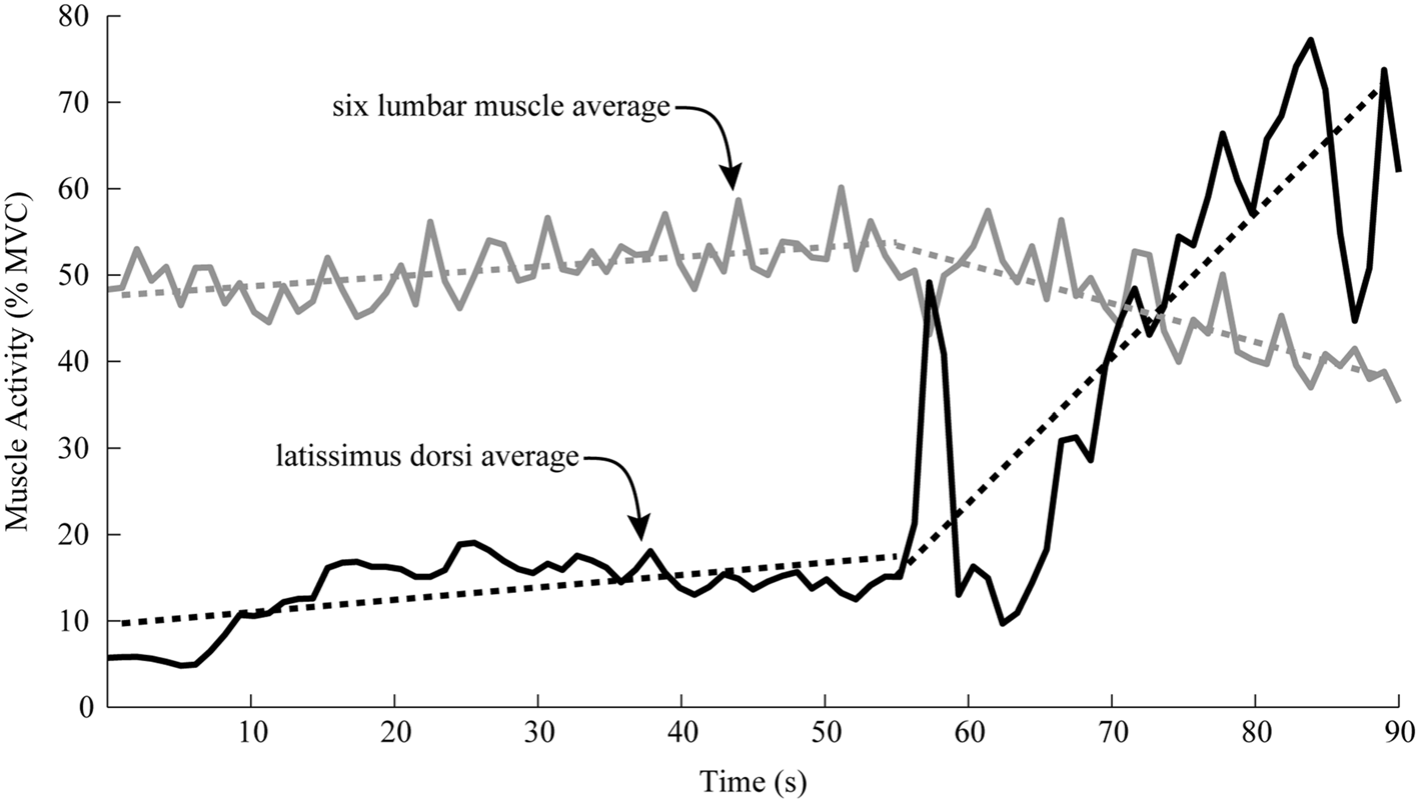
Latissimus dorsi (LD) and lumbar muscle interaction. Representative normalized muscle activity (RMS magnitude in %MVC, *N* = 1) of the LD muscle group (black line, mean of left and right LD) and the muscle activity of the lumbar muscles (gray lines, mean of all six recorded lumbar muscles) during a leaning trial (i.e., trial A2). Dashed lines are linear regression fits of each group mean during two distinct phases, i.e., phase 1 (0–60 s) where LD amplitude remains low, and phase 2 (60–90 s) where the LD amplitude increases substantially. The extension torque applied by the LD muscles about the lumbar spine can become significant (phase 2, increase in dashed-black slope) reducing the effort required by the low back muscles (phase 2, negative slope of dashed-gray line).

**Figure 8:**
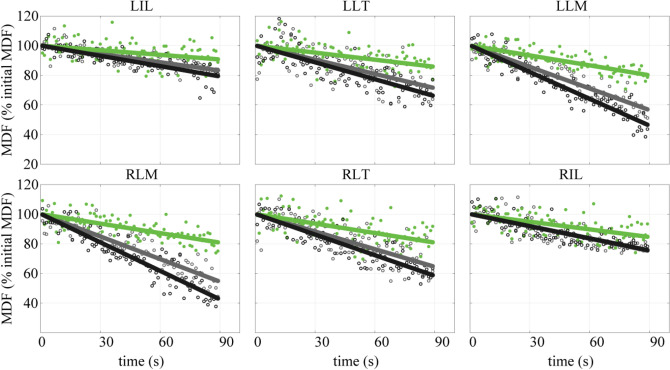
Subject 1, individual muscle MDF vs. time. Figure layout is identical to Fig. 3 in the main text.

**Figure 9:**
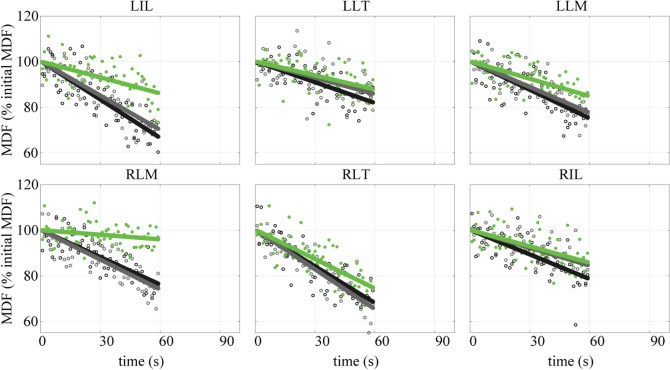
Subject 2, individual muscle MDF vs. time. Figure layout is identical to Fig. 3 in the main text.

**Figure 10:**
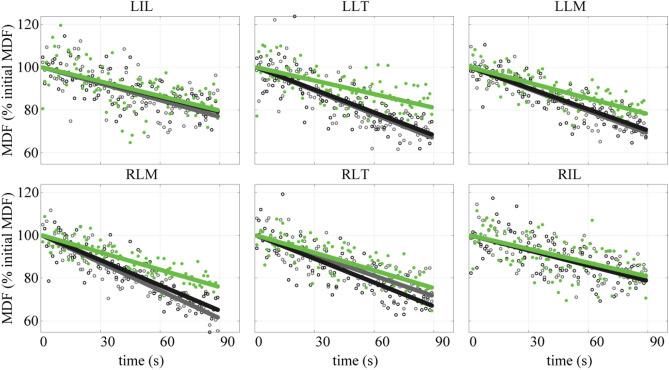
Subject 3, individual muscle MDF vs. time. Figure layout is identical to Fig. 3 in the main text.

**Figure 11:**
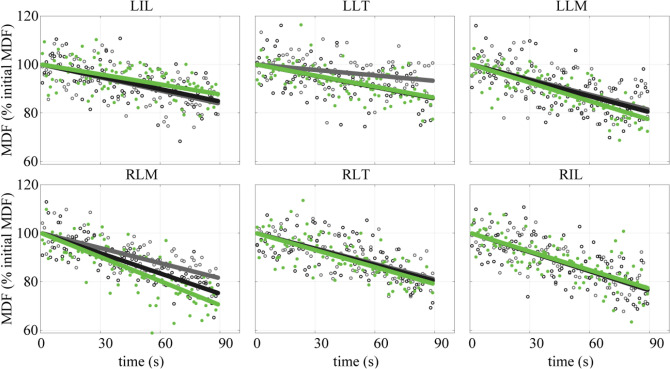
Subject 4, individual muscle MDF vs. time. Figure layout is identical to Fig. 3 in the main text.

**Figure 12:**
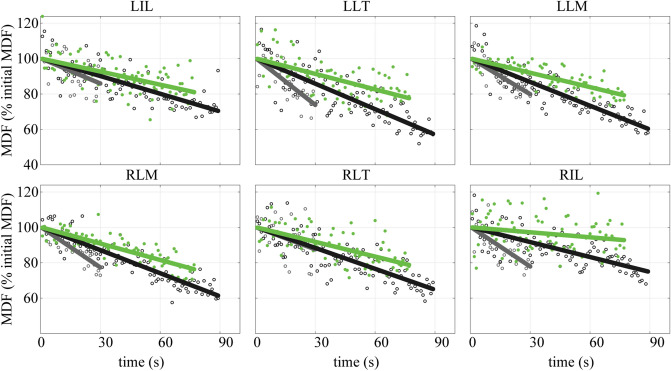
Subject 5, individual muscle MDF vs. time. Figure layout is identical to Fig. 3 in the main text.

**Figure 13:**
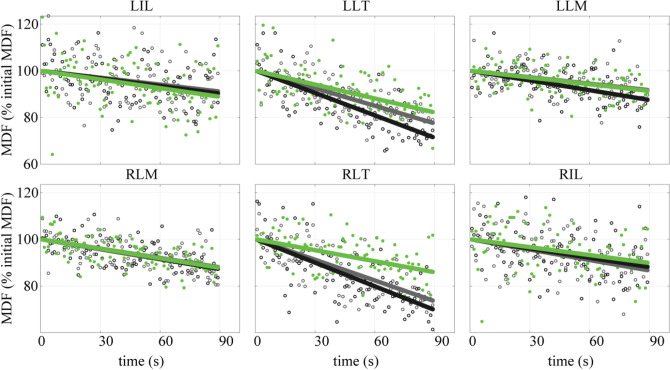
Subject 6, individual muscle MDF vs. time. Figure layout is identical to Fig. 3 in the main text.

We observed that the LD muscles could significantly affect the activity of the six lumbar muscles. The LD muscles primarily adduct, extend and medially rotate the shoulder joint, but they also contribute to lumbar extension. Previous studies have suggested that the magnitude of lumbar extension torque provided by the LD is generally small (and often negligible) relative to the contributions of other lumbar muscles during tasks such as lifting^27,40,41^. However, during pilot testing of the leaning task, we observed that some individuals would greatly increase their LD activity (typically about 60 s into the trial, Fig. 7). Concurrently, muscle activity from the six lumbar muscles would noticeably decrease; suggesting that subjects were offloading the six lumbar muscles by activating their LD muscles. Interestingly, this behavior of the LD muscles is somewhat similar to the behavior of the exosuit, because they both apply forces across the entire thoracolumbar spine, they both have a mechanical advantage relative to the underlying lumbar muscles (i.e., larger moment arm about the lumbar spine), and when engaged they can both offload other lumbar extensor muscles. Because of this LD behavior (which was employed inconsistently by subjects) we implemented a real-time visual feedback system (as detailed in Methods) to help minimize LD activity that would otherwise confound our interpretation of muscle fatigue rate based on conventional metrics (i.e., fitting a single linear regression to MDF data).

### Limitations

These findings should be considered and interpreted with a few experimental limitations in mind. The number of subjects tested with the exosuit intervention (*N* = *6*) was relatively small, resulting in low-power group-level (inter-subject) results. However, our main goal was to investigate responses at a subject-specific level. Using an A-B-A (withdrawal design) protocol provided convincing evidence that the exosuit assistance reduced the rate of lumbar muscle fatigue, and that the fatigue rate increased back to the baseline when assistance was removed (Table 2, Fig. 5). The magnitude of exosuit assistance varied between subjects (2.0–3.0 N/kg, Table 3). This variability was the result of prototype fit, manual tuning and other practical factors. Although this variability was not intentional, it revealed how the magnitude of fatigue reduction may be related to the magnitude of assistance, which is consistent with intuition and prior literature. On a related note, differences in body-type, anthropometry and muscle fiber composition could explain some variability observed between individual subjects and muscles; and these factors may be of interest to explore in future investigations. We did not include dynamic movements (such as lifting) in this study. For the simple leaning task explored in this work, we needed a highly controlled setup (e.g., custom scaffold and real-time feedback). The protocol would need to be altered to test dynamic movements. Nevertheless, in previous work we showed that this exosuit design can reduce lumbar muscle activity during static leaning as well as during dynamic movements like lifting, and this matches subjective feedback on perceived exertion while wearing the exosuit^13^. Also, prior studies using a somewhat similar back assistive device have demonstrated reductions in back muscle fatigue rate during lifting^10^. We measured sEMG over numerous sites (12, if we include abdominal and LD muscles); however, this still only represents a subset of muscles that contribute to lumbar flexion/extension. On a related note, we did not estimate contributions from passive (e.g., ligament) tissues around the spine; instead we attempted to keep these contributions small and consistent by targeting a neutral lumbar angle and by keeping this angle consistent across trials using biofeedback. It is important to note that only half of subjects tested during the Day 1 session showed reductions in MDF slope between the two weight conditions (11 kg vs. 16 kg). This highlights the importance of the validation trials in the design of muscle fatigue experiments which helped ensure for each subject tested that changes in muscle fatigue rates could be reliably and objectively measured with sEMG. Without subject-by-subject validation, the results may be difficult to interpret. One potential explanation for this finding is that the 5 kg difference between the two conditions (11 kg vs. 16 kg) may not have caused a large enough change in muscle activation for some subjects to exhibit changes in MDF slope; reflecting potential limitations in the sensitivity of this metric. Inducing greater changes in muscle activation would likely elicit more distinct changes in MDF slope (e.g., 10 kg difference in Farina et al.^18^). We note that non-parametric statistical tests are generally recommended for single-subject analysis, as detailed in Fisch^32^. But in this study, we used a parametric test (analysis of covariance) for the supplementary statistics. We used a parametric test because the accepted measure of sEMG muscle fatigue is based on linear regression, which is a parametric regression; thus, we could not perform non-parametric statistics without fundamentally altering the accepted measure of muscle fatigue (MDF slope from linear regression). Nevertheless, we have confidence in our findings because they were corroborated by visual inspection (Fig. 3, Appendix) and because the muscle fatigue rate during A2 returned back to the A1 baseline after the exosuit assistance was removed (Fig. 5, Table 2). Finally, asking subjects to lean without increasing activation of their latissimus dorsi adds an additional constraint which does not exist in typical work environments, but we found that it was necessary to control latissimus dorsi activity for conventional fatigue metrics to be valid and interpretable (i.e., applying linear regression to MDF data). We do not believe any of these limitations alter the overall conclusions from this study; the A-B-A protocol provides unambiguous evidence of reduced lumbar muscle fatigue in five of the six subjects when wearing the exosuit.

## Conclusion

We found that an un-motorized, low-profile elastic exosuit could significantly reduce the rate of lumbar muscle fatigue during leaning. We observed inter-subject variability in the number of muscles that benefited, which specific muscles benefited, and the degree to which each muscle benefited (percentage reduction in fatigue rate) from wearing the exosuit prototype. These findings suggest that this type of exosuit could potentially reduce the lumbar fatigue of individuals who perform bouts of leaning or bending (e.g., dentists, nurses, assembly line workers, material handlers and construction workers).

## Appendix

This Appendix depicts MDF vs. time results for each individual subject during Day 2 exosuit tests. The decrease in median frequency (MDF) over the duration of leaning is fit by linear regression lines. The slope of the regression line (i.e., MDF slope) is commonly used as a measurement of the rate of muscle fatigue^31^. Data are plotted for each muscle and each trial condition; control trial A1 (black line, open black circles), intervention trial B (green line, shaded green circles), control trial A2 (gray line, open gray circles). See Figs. 8, 9, 10, 11, 12, 13.
